# Supplementary figures and images for: Transcriptome and metabolomics analysis of adaptive mechanism of Chinese mitten crab (Eriocheir sinensis) to aflatoxin B1
Source: PLoS One. 2023 Dec 7;18(12):e0295291. doi: 10.1371/journal.pone.0295291 (PMC10703319; doi:10.1371/journal.pone.0295291)

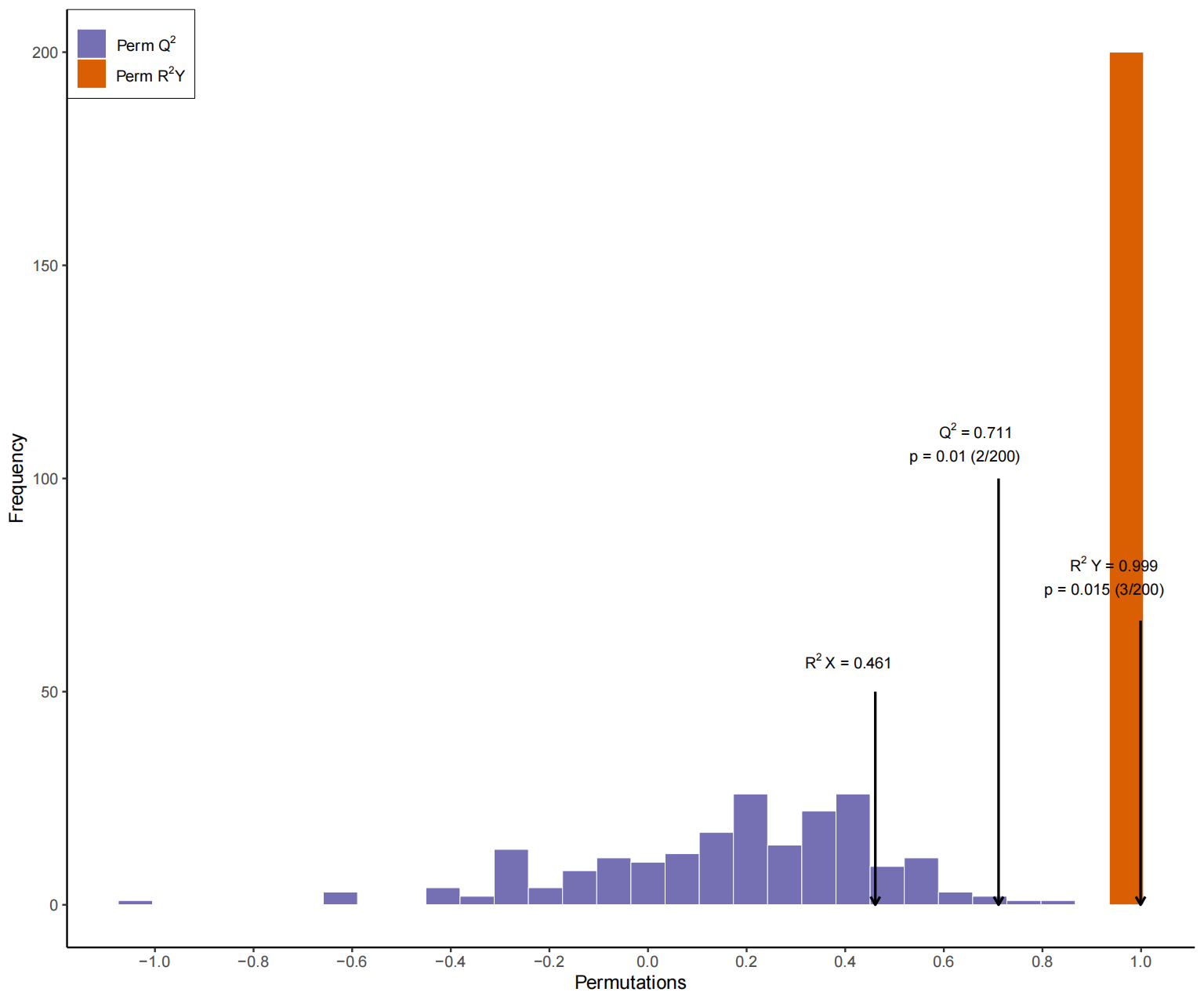

Supplement: S1 Fig — (TIF) [file pone.0295291.s001.tif]

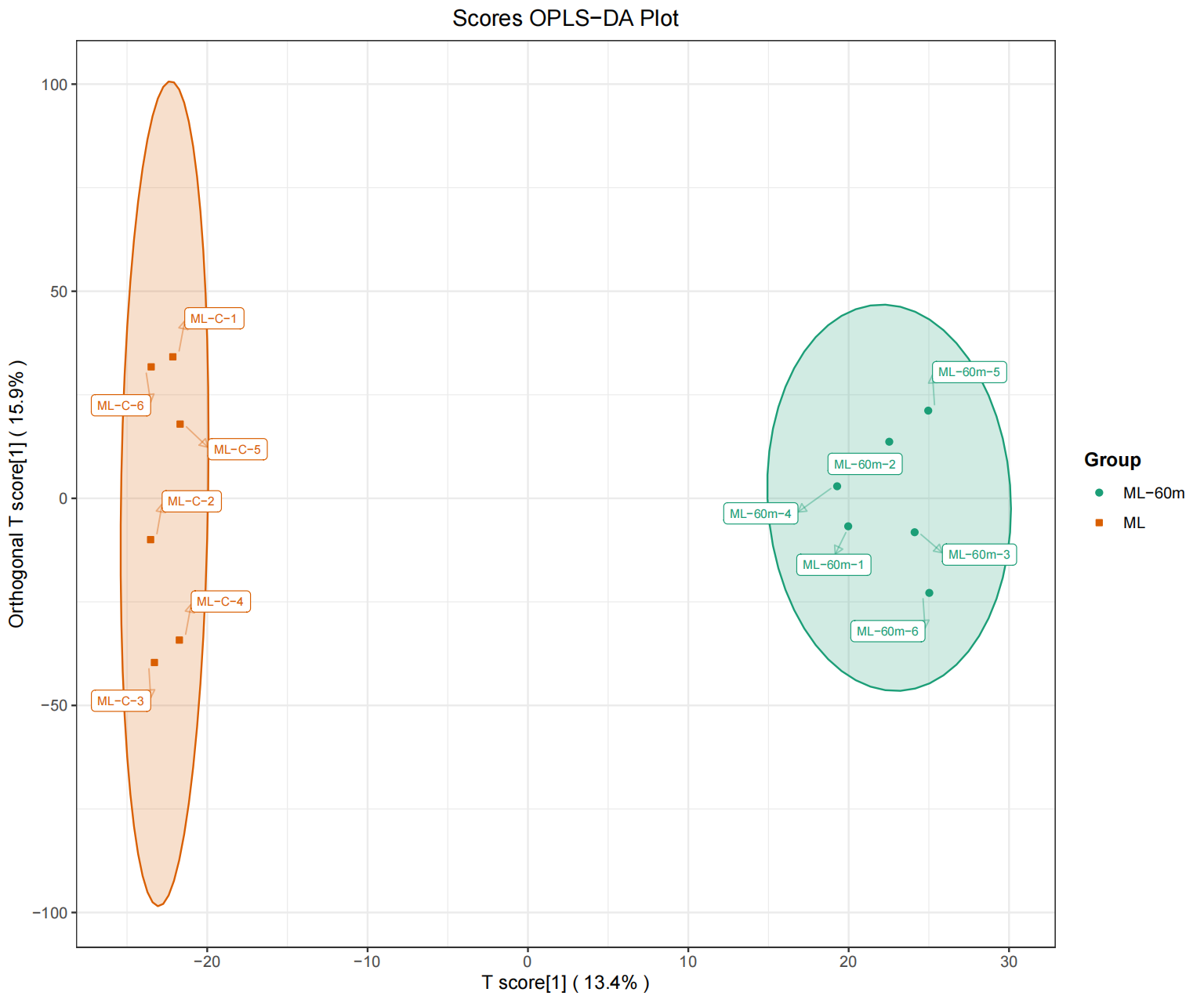

Supplement: S2 Fig — (TIF) [file pone.0295291.s002.tif]
