# Supplementary material for: Transcriptome and metabolomics analysis of adaptive mechanism of Chinese mitten crab (Eriocheir sinensis) to aflatoxin B1
Source: PLoS One. 2023 Dec 7;18(12):e0295291. doi: 10.1371/journal.pone.0295291 (PMC10703319; doi:10.1371/journal.pone.0295291)
Supplement: S3 Table — (DOCX) [file pone.0295291.s005.docx]

Table 4 The cored and up-regulated DEGs during AFB1 injection in hepatopancreas

| Gene ID | Gene name | NR annotation | GO term | Function |
| --- | --- | --- | --- | --- |
| LOC126983756 | UNC119 | protein unc-119 homolog B-like | lipid binding;nervous system development | a photoreceptor synaptic protein, the photoreceptor synapses in the outer plexiform layer of the retina. |
| LOC127004138 | _ | anti-lipopolysaccharide factor 2 | _ | an important antibacterial peptide of shrimp, it plays an important role in the process of removing infected foreign pathogens. |
| LOC126992081 | BAIAP3 | Protein unc-13 4B | _ | regulate neurotransmitter release |
| LOC127005812 | SCP2,SCPX | non-specific lipid-transfer protein | lipid binding; integral component of membrane; CoA C-acyltransferase activity; peroxisome; lipid transport | facilitate uptake and metabolism and detoxification of dietary-derived phytol in mammals |
| LOC126981104 | TYR | hemocyanin subunit 6 | oxidoreductase activity | copper-containing respiratory proteins; oxidoreductase activity |
| LOC127005969 | PRSS1_2_3 | trypsin-like serine proteinase | serine-type endopeptidase activity;integral component of membrane | physiological and pathological processes such as blood coagulation, immune response, fibrinolysis, angiogenesis, inflammation and tumor |
| LOC126986919 | ARSB | arylsulfatase B-like | sulfuric ester hydrolase activity | Sulfate metabolism |
| LOC127004133 | _ | juvenile hormone esterase; Venom carboxylesterase-6 | carboxylic ester hydrolase activity | Eliminate inflammation |
| LOC126981425 | SARDH | sarcosine dehydrogenase, mitochondrial-like | oxidoreductase activity | oxidoreductase activity |
| LOC126983580 | TMEM189 | transmembrane protein 189-like | fatty acid metabolic process | fatty acid metabolic process |
| LOC126981590 | IDH1,IDH2 | Isocitrate dehydrogenase [NADP] cytoplasmic | tricarboxylic acid cycle;magnesium ion binding;isocitrate dehydrogenase (NADP+) activity;NAD binding;glyoxylate cycle;isocitrate metabolic process | ATP synthesis provides energy for biological activities |
| novel.4226 | RPP40 | putative RNA-directed DNA polymerase from mobile element jockey-like | RNA-directed DNA polymerase activity | High value of DNA polymerase indicates deterioration of liver function |
| LOC126997672 | _ | Copper-specific metallothionein-2 | metal ion binding | Fixation and preservation of heavy metal elements and detoxification of ionic heavy metals |
| LOC126998993 | SORD,gutB | Sorbitol dehydrogenase | zinc ion binding;oxidoreductase activity;L-iditol 2-dehydrogenase activity;sorbitol catabolic process | It strongly suggests liver damage, and serum SDH can reflect liver damage |
