# Supplementary material for: Transcriptome and metabolomics analysis of adaptive mechanism of Chinese mitten crab (Eriocheir sinensis) to aflatoxin B1
Source: PLoS One. 2023 Dec 7;18(12):e0295291. doi: 10.1371/journal.pone.0295291 (PMC10703319; doi:10.1371/journal.pone.0295291)
Supplement: S7 Table — (DOCX) [file pone.0295291.s009.docx]

Table 8 KEGG pathways were Co-enriched by DEGs and DAMs in C vs. 30 m comparison

| KEGG_map | Description | Index_meta | CID_meta | Index_gene | KO_gene |
| --- | --- | --- | --- | --- | --- |
| ko00100 | Steroid biosynthesis | MW0148978 | C05440 | LOC126988274;  LOC127008729 | K01052;  K00227 |
| ko00480 | Glutathione metabolism | MW0106760;MW0110603 | C03740;  C01672 | LOC126981512;  LOC126981590;  LOC127008402 | K11140;  K00031;  K00033 |
| ko00260 | Glycine, serine and threonine metabolism | MW0106578 | C06231 | LOC126981425;  LOC127008985 | K00314;  K00306 |
| ko00380 | Tryptophan metabolism | MW0169364;MW0123335 | C01717;  C05640 | LOC127009255 | K00452 |
| ko00350 | Tyrosine metabolism | MW0003626;MEDN0648 | C05580;  C05585 | LOC126981104 | K00505 |
| ko00310 | Lysine degradation | MW0110603;MW0014566 | C01672;  C01181 | LOC127008985 | K00306 |
| ko00230 | Purine metabolism | MW0103590;MEDN1006 | C00144;  C00366 | LOC126999937 | K01587 |
