# Supplementary material for: Transcriptome and metabolomics analysis of adaptive mechanism of Chinese mitten crab (Eriocheir sinensis) to aflatoxin B1
Source: PLoS One. 2023 Dec 7;18(12):e0295291. doi: 10.1371/journal.pone.0295291 (PMC10703319; doi:10.1371/journal.pone.0295291)
Supplement: S8 Table — (DOCX) [file pone.0295291.s010.docx]

Table 9 KEGG pathways were Co-enriched by DEGs and DAMs in C vs. 60 m comparison

| KEGG_map | Description | Index_meta | CID_meta | Index_gene | KO_gene |
| --- | --- | --- | --- | --- | --- |
| ko00350 | Tyrosine metabolism | MW0003626;MEDN0648;  MW0004236 | C05580;  C05585;  C05587 | LOC126988775;  LOC126988766;  LOC126981097;  LOC126981104;  LOC126994668;  LOC126988767;  LOC127005152 | K00505;  K00505;  K00505;  K00505;  K00815;  K00505;  K15849 |
| ko02010 | ABC transporters | MW0159913;MEDP0143 | C00294;  C00120 | LOC126993488;  LOC127007428 | K05643;  K05673 |
| ko00564 | Glycerophospholipid metabolism | MW0056883;MW0054553 | C00157;  C04230 | LOC126981855;  LOC127002076 | K14286;  K01115 |
| ko00061 | Fatty acid biosynthesis | MW0054288 | C02679 | LOC127007862 | K01897 |
| ko01040 | Biosynthesis of unsaturated fatty acids | MEDL02002;  MEDL00450 | C08316;  C00219 | LOC127005812 | K08764 |
| ko00380 | Tryptophan metabolism | MEDL02773 | C00637 | LOC126999000 | K03392 |
| ko00260 | Glycine, serine and threonine metabolism | MW0107125 | C00581 | LOC126981425 | K00314 |
| ko00600 | Sphingolipid metabolism | MW0111287;MEDP1685;  MW0055323;MW0011289 | C00836;  C12144;  C00550;  C06125 | LOC126997840 | K01201 |
| ko00230 | Purine metabolism | MW0159913;MW0103590;MEDN1006 | C00294;  C00144;  C00366 | LOC126982181 | K01466 |
