# Supplementary material for: Transcriptome and metabolomics analysis of adaptive mechanism of Chinese mitten crab (Eriocheir sinensis) to aflatoxin B1
Source: PLoS One. 2023 Dec 7;18(12):e0295291. doi: 10.1371/journal.pone.0295291 (PMC10703319; doi:10.1371/journal.pone.0295291)
Supplement: Attachment — Submitted filename: Manuscript SSE3092023.docx [file pone.0295291.s011.docx]

**Transcriptome and metabolomics analysis of adaptive mechanism of Chinese mitten crab (Eriocheir sinensis) to aflatoxin B1**

Hongsheng Yang^1,2†^, Meifang Shen^1^, Qiuyun Zhang^2^, Yifeng Li^3^, Xiuhui Tan^2^, Xuguang Li^2^, Huimin Chen^4^ , Lei Wu^5^, Shaofang He^5^, Xiaohua Zhu^1*^

1. Fishery analysis and Testing Center of Jiangsu Province, Nanjing, 210017, China

2. Freshwater Fisheries Research Institute of Jiangsu Province, Nanjing 210017, China

3. College of Aquatic and Life Sciences, Shanghai Ocean University, Shanghai 201306, China

4. SCIEX Analytical Instrument Trading Co., Shanghai 200335, China

5. Yitian Technologies Corporation, Nanjing, 210095, China.

^*^Correspondence:

Xiaohua Zhu, Fishery analysis and Testing Center of Jiangsu Province, Nanjing, China.

Email: xhz824@sina.com

**Abstract**

Aflatoxin B1 (AFB1), with the strong toxicity and carcinogenicity, has been reported to great toxicity to the liver and other organs of animals. It cause huge economic losses to breeding industry, including the aquaculture industry. Chinese mitten crabs (Eriocheir sinensis), as one of important species of freshwater aquaculture in China, are deeply disturbed by it. However, the molecular and metabolic mechanisms of hepatopancreas and ovary in crabs underlying coping ability are still unclear. Hence, we conducted a targeted injection experiment with or without AFB1, and comprehensively analyzed transcriptome and metabolomics of hepatopancreas and ovary. As a result, 210 and 250 DEGs were identified in the L-C vs. L-30 m and L-C vs. L-60 m comparison, among which 14 common DEGs were related to six major functional categories, including antibacterial and detoxification, ATP energy reaction, redox reaction, nerve reaction, liver injury repair and immune reaction. A total of 228 and 401 DAMs in the ML-C vs. ML-30 m and ML-C vs. ML-60 m comparison both enriched 12 pathways, with clear functions of cutin, suberine and wax biosynthesis, tyrosine metabolism, purine metabolism, nucleotide metabolism, glycine, serine and threonine metabolism, ABC transporters and tryptophan metabolism. Integrated analysis of metabolomics and transcriptome in hepatopancreas discovered three Co-enriched pathways, including steroid biosynthesis, glycine, serine and threonine metabolism, and sphingolipid metabolism. In summary, the expression levels and functions of related genes and metabolites reveal the regulatory mechanism of Chinese mitten crab (Eriocheir sinensis) adaptability to the Aflatoxin B1, and the findings contribute to a new perspective for understanding Aflatoxin B1 and provide some ideas for dealing with it.

**Introduction**

Aflatoxin is a secondary metabolite produced by Aspergillus flavus and Aspergillus parasiticus, and is a highly toxic natural pollutant widely present in food and feed in humid and hot areas〔1-2〕. Among the 12 aflatoxin derivatives detected, the most toxic and carcinogenic aflatoxin B1 (AFB1) is the most common, with significant toxicity to the liver and other organs of animals〔3〕.

In recent years, aflatoxin pollution has caused huge economic losses to breeding industry, including aquaculture industry〔4〕. According to data from the Food and Agriculture Organization (FAO) of the United Nations, approximately 25% of crops worldwide are contaminated with varying degrees of aflatoxin every year, and about 2% of crops lose their feed value due to severe pollution〔5,6〕.

The research on the impact of AFB1 on aquatic animals at home and abroad is mainly focused on the Oreochromis niloticus [7,8], Oncorhynchus mykiss [9], Litopenaeus vannamei [10], Lctalurus punctatus [11], Labeo rohita [12] and other species. According to reports, due to feeding Oreochromis niloticus with AFB1 for 10 weeks, the growth rate slows down and the mortality rate gradually increases [13]. The reproductive capacity of Carassius auratus gibelio fed with AFB1 is severely affected [14]. Current research shows that AFB1 can cause abnormal behavior, slow growth, immunosuppression, antioxidant damage, and tissue lesions of aquatic animals [15-17]. If AFB1 accumulates in the body tissues of aquatic animals and spreads further in the ecosystem along with the food chain, it may threaten human health [18]. Therefore, the agricultural industry standard of the People's Republic of China (NY5072-2002) stipulates that the safety limit of AFB1 in fishery composite feed is 10μg/kg [19].

Chinese mitten crab (Eriocheir sinensis) is one of the important species in freshwater aquaculture in China, which has produced good economic benefits [20]. In recent years, with the continuous expansion of aquaculture scale and the continuous improvement of intensification, various diseases have emerged in Chinese mitten crab, seriously hampering its healthy development [21]. As mentioned above, we are aware that AFB1 has significant toxicity to animal livers [3]. Therefore, in this experiment, the crab hepatopancreas is the main research organ, and we hope to obtain some data to explore the molecular response mechanism. The 400 uL aflatoxin B1 of standard solution was injected into the tested crabs, and the experimental data of hepatopancreas and ovary within one hour after treatment were obtained through transcriptome sequencing and metabonomics methods. The results of this experiment provide data support for our study on the stress response of aflatoxin B1 to crabs, and provide ideas for solving the practical production problems of aflatoxin B1 on crabs in the future.

**Materials and methods**

**Experimental reagent**

The aflatoxin B1 standard, with a purity of 99.8%, was provided by Israel FERMENTEK Company. The Dimethyl sulfoxide (chromatographically pure) was provided by ROE Company of the United States. The standard AFB1 working solution, with a concentration of 60 mg/L, was prepared by weighing a certain amount of aflatoxin B1 standard and dissolving it with dimethyl sulfoxide. The blank working solution was prepared using a single dimethyl sulfoxide reagent.

**Experimental Design and Sample Collection**

A certain amount of female experimental crabs were raised at the Pukou Breeding Base in Nanjing, Jiangsu Province, China. Before the experiment, 18 female crabs with an initial average weight of (125±12g) were randomly divided into three groups. Two groups of female experimental crabs were injected with disposable needles for 400 uL AFB1 standard solution with a concentration of 60 mg/L, and the third group was injected with 400 uL blank working solution. The hepatopancreas and ovaries of experimental crabs injected with AFB1 were taken out after 30 minutes or 60 minutes, respectively. The hepatopancreas and ovaries of experimental crabs injected with blank working solution were taken out as control after 30 minutes. Frozen samples with liquid nitrogen, then stored in an ultra-low temperature refrigerator (-80**℃**). Six samples in each group were used for metabolomics, and three samples in each group were randomly selected for transcriptome sequencing.

**RNA Extraction and Transcriptome sequencing**

Total RNA was extracted from frozen samples using a Trizol reagent method (DP762-T1C). RNA purity and integrity were assessed by a NanoPhotometer® spectrophotometer (IMPLEN, CA, USA) and a RNA Nano 6000 Assay Kit on the Agilent Bioanalyzer 2100 system (Agilent Technologies, CA, USA). RNA contamination and degradation were assessed by 1.5% agarose gel electrophoresis.

A total of 1 μg of RNA per sample was used as the input material for library preparation. The mRNA was purified from the total RNA using poly-T oligo-attached magnetic beads. Sequencing libraries from different groups were constructed with the purified mRNA using the NEBNext® UltraTM RNA Library Prep Kit for Illumina® (NEB, USA). According to the manufacturer’s recommendations, the cDNA library preparations for next-generation sequencing were generated from each sample. Each cDNA library was purified with AMPure XP system (Beckman Coulter, Beverly, MA, USA) and its quality was assessed by using Agilent RNA 6000 Nano Kit on the Agilent Bioanalyzer 2100 system (Agilent Technologies, Santa Clara, CA, USA). The index codes were added to attribute sequences to each sample, and the cDNA libraries constructed by the above reaction system were performed on an Illumina HiSeq X Ten platform by Frasergen Bioinformatics Co., Ltd. (Wuhan, China). As a result, 150-bp paired-end reads were generated and then entered the filtering quality control process.

Processed raw reads and aligned clean reads to the reference genome Chinese mitten crab (Eriocheir sinensis) (https://www.ncbi.nlm.nih.gov/assembly/GCF_024679095.1) using HISAT 2 software [22]. Use Sam tools to compress and sort mapping files, and use String Tie software for read assembly and abundance estimation [23]. In this experiment, the fragments per kilo base of exon per million fragments mapped reads (FPKM) represented the relative expression of genes, and FDR was a prerequisite for determining whether it was a differentially expressed gene (DEG). The list of DESeq2 identifies DEGs, which was filtered with a conservative absolute FC (fold change) cutoff of at least 2 and a cutoff for multiple testing corrected p-value (FDR) of at most 0.05. GO (Gene Ontology) and KEGG (Kyoto Encyclopedia of Genes and Genomes) pathway enrichment was performed using phyper R package.

**Metabolic Profiling**

100 mg powdered samples were homogenized and were extracted with 1.0 mL pre-chilled 80% methanol solution containing 0.1% (v/v) formic acid at 4 °C overnight. The extracts were centrifuged for 10 min at 4 ◦C, 10,000 g, then absorbed and diluted into a concentration containing 53% methanol. The supernatant was filtrated before LC-ESI-MS/MS system (HPLC, Shim-pack UFLC SHIMADZU CBM30A system; MS, Applied Biosystems 4500 Q TRAP). LC–MS/MS analyses were performed using an ExionLCTM AD system coupled with a QTRAP® 6500+ mass spectrometer (both from SCIEX, Framingham, MA, USA). It described the system including the high performance liquid chromatography (HPLC) and MS conditions by He et al [24].

Identification and quantification of metabolites were analyzed using the MultiaQuant and Analyst software (1.6.1). The multivariate analysis of metabolites, including model evaluation, was conducted by the orthogonal partial least squares discriminant analysis (OPLS-DA) with score plots and permutation plots. Differential metabolites between sample groups were estimated by the absolute value of Log2FC ≥1, P value < 0.05, as well as the variable importance in the projection (VIP) ≥ 1 using the R package MetaboAnalystR. The identified metabolites were aligned to GO and KEGG database, and the significantly enriched functions explained the differences between sample groups.

**Results**

**Transcriptome (RNA-Seq) Analysis**

Data acquisition of Transcriptome

In this study, transcriptome data of hepatopancreas (L) and ovary (R) were statistically analyzed. A total of 18 experimental samples generated over 148.12 Gb of clean data. The clean data from each sample reached at least 6.08 Gb and the percentage of Q30 bases was 93.90% and above (Table 1). Mapping ratio between clean reads and the reference genome of Eriocheir sinensis (https://www.ncbi.nlm.nih.gov/assembly/GCF_024679095.1) ranged from 80.27 to 88.34%, with an average of 84.86% (Table 1). Principal component analysis (PCA) showed significant differences between the untreated group (C) and the treat groups (30 m, 60 m) in hepatopancreas and ovary, respectively. The changes after treatment were not uniform, and the repeatability within each group was acceptable. PC1 (40.44%) and PC2 (25.09%) effectively separated the control samples from the treatment samples in hepatopancreas (Fig1a), and PC1 (39.64%) and PC2 (24.14%) separated the control samples from the treatment samples in ovary (Fig1a). The above results demonstrated that the processing method and the data obtained could be used.

Analysis of Differentially expressed genes

Differentially expressed genes (DEGs) defined by fold change (FC) ≥ 2 and FDR < 0.01 reflected the gene expression patterns between different sample groups, therefore, providing some explanations for the body's response within 1 hour of AFB1 stress. Groups using blank working solution (L-C) in hepatopancreas compared with groups using standard AFB1 working solution (L-30 m and L-60 m), as well as groups using blank working solution (R-C) in ovaries compared with groups using a standard AFB1 working solution (R-30 m and R-60 m). Specifically, we performed the conventional analysis of the pairwise comparisons of C vs. 30 min and C vs. 60 min in hepatopancreas and ovaries, respectively. Results showed, 210 (133 up-regulated; 77 down-regulated) and 250 (140 up-regulated; 110 down-regulated) DEGs were identified in the L-C vs. L-30 m and L-C vs. L-60 m comparison in hepatopancreas, respectively. Further analysis revealed 64 DEGs were the same in L-C vs. L-30 m and L-C vs. L-60 m comparison, with 34 DEGs up-regulated and 30 DEGs down-regulated (Fig 1b, Table2, 3). The 34 common up-regulated DEGs mentioned above had attracted our attention, guiding us to understand which genes were more active and which functions were enhanced after stress (Fig 1b). Studies had found that 14 up-regulated DEGs showed relatively complete functional annotation information or biological function related to this study (Table 4). Based on the relevant gene annotations and functional gene research reports in the literature, we had simply classified the above 14 DEGs into six categories of functions, including antibacterial and detoxification, ATP energy reaction, redox reaction, nerve reaction, liver injury repair, and immune reaction. Among them, five DEGs were related to antibacterial and detoxification, followed by two DEGs related to ATP energy reaction, redox reaction, nerve reaction, and liver injury repair, and one DEG related to immune reaction (Fig 2). The log2FC values of 14 DEGs ranged from 1.35 to 21.11, and the log2FC values of same gene between L-C vs. L-30 m and L-C vs. L-60 m was not significantly different, indicating that the possibility of individual differences leading to differential genes was very small (Fig 2). Literature showed that gene LOC127005969 (PRSS1_2_3) encoded trypsin-like serine proteinase and played an important role in the repair of pathological processes such as coagulation, immune response, fibrinolysis, inflammation and tumor [25]. The log2FC values of gene LOC127005969 were 5.18 (L-C vs. L-30 m) and 4.83 (L-C vs. L-60 m), showing a significant increase in the treated group compared with the untreated group. Gene LOC126998993 (SORD) encoded sorbitol dehydrogenase, and high expression of this protein indicated a signal of liver injury [26]. The expression level of LOC126998993 increased nearly fourfold before and after treatment (the log2FC values of 1.96 in L-C vs. L-30 m and 1.77 in L-C vs. L-60 m, respectively). Gene LOC127004138 could encode anti-lipopolysaccharide factor, which was widely used in aquaculture due to its strong antibacterial activity and broad antibacterial spectrum [27]. The expression level of LOC127004138 increased more than fourfold before and after treatment (the log2FC values of 2.12 in L-C vs. L-30 m and 2.84 in L-C vs. L-60 m, respectively). Additionally, the increased expression of several proteases or enzymes reflects the enhanced detoxification ability of hepatopancreas to cope with the invasion of AFB1, such as lipid-transfer protein encoded by gene LOC127005812 (SCP2); arylsulfatase encoded by gene LOC126986919 (ARSB); hormone esterase encoded by gene LOC127004133 and copper-specific metallothionein encoded by gene LOC126997672. All these proteins had been reported to be associated with detoxification [28], and in this study, the expression levels of these genes in the treated group increased by at least two times compared to the untreated group. In addition to these genes mentioned above, we also studied and analyzed other biological activities, such as redox reactions, neural reactions, and energy activities.

In the ovaries, 124 (45 up-regulated; 79 down-regulated) and 99 (68 up-regulated; 31 down-regulated) DEGs were identified in the comparison of R-C vs. R-30 m and R-C vs. R-60 m, respectively (Fig 3a). The 124 and 99 DEGs mentioned above have a total of 12 shared DEGs, of which 7 DEGs were up-regulated and 5 DEGs were down-regulated (Fig 3b, Table 5). NR annotation indicated most of DEGs were associated with proteins that have not been characterized or hypothesized. Only four genes (LOC127003043; novel.27046; novel.898; LOC127005871) were annotated in GO terminology, and GO explained the function of these genes as integral component of membrane. In the experiment on the ovaries, there were no characteristic DEGs presented, which reflected that within 1 hour of AFB1 invasion, the stress response activity of the ovaries was much less intense than that of the hepatopancreas.

K-means analysis in hepatopancreas

To decipher the overall trend in gene expression profiles, k-means clustering method was applied to determine the trend change of DEGs in hepatopancreas. We subjected 318 DEGs in hepatopancreas to k-means clustering process and obtained five distinct subclusters (Fig 4). As a result, subcluster 2 revealed that the expression levels of 49 DEGs decreased after 30 minutes and remained basically unchanged from 30 minutes to 60 minutes. Subcluster 3 revealed the expression levels of 61 DEGs continued to increase from the beginning to 60 minutes. The expression levels of 44 DEGs showed a trend of first decreasing and then increasing from 30 minutes to 60 minutes (subcluster 1), while 23 DEGs first increased and then decreased from 30 minutes to 60 minutes (subcluster 5).

**Metabolomics Analysis**

Analysis of metabolites based on UPLC-MS/MS-Based Quantitative

To further explore the reactions in hepatopancreas and ovary within 1 hour after injection of AFB1 standard solution, an untargeted metabolomics method were used to detect metabolite compounds and investigate differential metabolites produced in the pairwise comparisons. In this study, a total of 13,963 metabolites were detected or identified, of which 6,642 were positive model and 7,321 were negative model. The number of metabolites for secondary identification was 4,829 in positive model and 4,003 in negative model, so we selected metabolites from positive model for subsequent analysis. Statistical analysis revealed that the components of metabolites were classified into 25 major categories, with amino acid and its metabolites accounting for 27.33%, followed by benzene and substituted derivatives (12.41%), and heterocyclic compounds (11.4%)(Fig 5a). The differential metabolites were searched by the model for orthogonal partial least squares discriminant analysis (OPLSDA), which performed 200 random permutations and combination experiments on the data. The OPLSDA results show that the model parameters for R2Y and Q2 values were close to 1, further verifying the reliability of the OPLSDA Model (Fig S1, 2).

Functional Annotation and Enrichment Analysis of Differential Metabolites

Combined with the multivariate analysis of OPLS-DA, the variable importance projection (VIP) > 1 together with fold change ≥2 or ≤0.5, was used as the screening criteria for differential abundant metabolites. In a hepatopancreatic positive model, group using the blank working solution (ML-C) was compared with groups using the standard AFB1 working solution (ML-30 m and ML-60 m), resulting the differentially accumulated metabolites (DAMs). The OPLS-DA model maximized the discrimination between group using the blank working solution (ML-C) and groups using the standard AFB1 working solution (ML-30 m and ML-60 m), showing significant differences (Fig 5b). In the comparison between ML-C and ML-30 m, a total of 228 metabolites (133 up-regulated and 95 down-regulated) showed significant differences. A total of 401 metabolites (339 up-regulated and 62 down-regulated) were identified as DAMs in the ML-C vs. ML-60 m comparison (Fig 5c). According to the functional annotation of DAM, based on the Kyoto Encyclopedia of Genes and Genomes (KEGG) database, 25 and 31 pathways were enriched in the comparison of ML-C vs. ML-30 m and ML-C vs. ML-60 m, respectively (Table 6,7). KEGG pathway analysis founded they both enriched a total of 12 pathways, with clear functions of cutin, suberine and wax biosynthesis; tyrosine metabolism; purine metabolism; nucleotide metabolism; glycine, serine and threonine metabolism; ABC transporters and tryptophan metabolism. The significant enrichment pathways (P<0.05) in comparison between ML-C and ML-30 m were cyanoamino acid metabolism (ko00460), glutathione metabolism (ko00480) and sulfur relay system (ko04122) (Fig 6a). More specifically, N-hydroxy-L-isoleucine (MW0154477) and N-hydroxy-L-valine (MW0154482), as members of amino acid and its metabolites, were DAMs in the ML-C vs. ML-30 m comparison and participated in the ko00460 pathway. In addition, gamma-glutamylalanine (MW0106760) and cadaverine (MW0110603) participated in the ko00480 pathway, and thiamine (MEDP0514) participated in the ko04122 pathway. In order to understand the synergistic or mutually exclusive relationship between different metabolites, the interactions between the top 50 differential metabolites were subjected to process by the Pearson correlation analysis method. The results showed that amino acid and its metabolites accounted for the highest proportion, with a total of 17 DAM members (Fig 6d).

The significantly enrichment pathways (P<0.05) in ML-C vs. ML-60 m comparison were sphingolipid metabolism (ko00600) (Fig 6b). Sphinganine(MW0111287),N-(dodecanoyl)-sphing-4-enine-1-phosphocholine (MW0055323) and phytosphingosine (MEDP1685), as members of sphingolipids (SL), were DAMs in ML-C vs. ML-60 m comparison and participated in the ko00600 pathway. The top 50 differential metabolites contained ten kinds of amino acid and its metabolites, seven kinds of fatty acyls (FA), and six kinds of benzene and substituted derivatives (Fig 6e).

In the ovarian positive model, group using the blank working solution (MR-C) in the ovaries was compared with groups using the standard AFB1 working solution (MR-30 m and MR-60 m), generating 262 (145 up-regulated and 117 down-regulated) and 452 (91 up-regulated and 361 down-regulated) differential metabolites in the comparison of MR-C vs. MR-30 m and MR-C vs. MR-60 m, respectively. In the comparison between MR-C and MR-30 m, no significant enrichment pathways were found in the 36 enriched KEGG pathways (P<0.05), while in the comparison between MR-C and MR-60 m, 10 significantly enriched KEGG pathways (P<0.05) were found in the 66 enriched KEGG pathways, including 2-oxocarboxylic acid metabolism, tropane, piperidine and pyridine alkaloid biosynthesis, glucosinolate biosynthesis, flavonoid biosynthesis, phenylalanine metabolism, aminoacyl-tRNA biosynthesis, D-amino acid metabolism, fatty acid biosynthesis, and cysteine and methionine metabolism (Fig 6c). The top 50 differential metabolites contained 11 kinds of benzene and substituted derivatives, followed by 10 kinds of amino acid and its metabolites (Fig 6f).

**Integrated Analysis of Metabolomics and Transcriptomics**

The comprehensive analysis of metabolomics and transcriptome could systematically study the coordinated response of crabs to AFB1 stress. The combined data of multiple genomics could further analyze the interaction between genes and metabolites, so as to analyze and deal with this stress response. According to the data of metabolomics and transcriptomics, the DEGs and DAMs derived from the comparisons of ML-C vs. ML-30 m and ML-C vs. ML-60 m were Co-enriched and analyzed. The results showed that in ML-C and ML-30 m, 11 DEGs and 12 DAMs, were Co-enriched into 7 pathways in KEGG enrichment pathways (Table 8); in the comparison between ML-C and ML-60 m, 17 DEGs and 19 DAMs Co-enriched into 9 pathways (Table 9). Among the 7 and 9 pathways mentioned above, there are four pathways, including glycine, serine and threonine metabolism, tryptophan metabolism, tyrosine metabolism and purine metabolism, which were shared by both (Fig 7a). In order to further explore the role of genes and metabolites in the same pathway, we anchored the up-regulated DEGs and DAMs at specific locations and studied their interactions. From certain positions in the three pathways of steroid biosynthesis (ko00100), glycine, serine and threonine metabolism (ko00260), and sphingolipid metabolism (ko00600), there were up-regulated DEG and DAM involved in co- regulation, which had caught our attention. In steroid biosynthesis (ko00100) pathway, enzyme sterols were products of transcription-translation and modification by gene LOC127008729 (EGR3), which catalyzed the conversion of episterol into ergostathenol (Fig 7b). Ergostathenol was converted to ergostatetraenol (C05440) under a action of the isoenzymes of EGR, and then into ergosterol. Ergosterol, as an important source of fat-soluble vitamin D2, had significant antibacterial and anti-tumor effects [29-30]. In this pathway, the mean FPKM of DEG EGR3 in the L-C group and L-30m group were 16.40 and 40.08, respectively. The expression level of DAM MW0148978 (C05440) significantly increased from ML-C stage to ML-30 m stage and showed differences. The second ko00260 pathway showed that DEG LOC126981425 (SARDH) encoded sarcosine dehydrogenase, which catalyzed of sarcosine to glycine (Fig 7c). Recent studies have found that the mechanisms of glycine included physiological protection against ischemia reperfusion injury, shock, transplantation, alcoholic hepatitis, liver fibrosis, arthritis, tumor, and drug toxicity, as well as anti-inflammatory and immunomodulatory effects [31-32]. A portion of glycine was involved in clyoxylate metabolism, and the other portion was converted to guanidinoacetate (C00581) under the action of GATM, ultimately converting to creatine and entering arginine and proline metabolism. Creatine contributed to the circulation of adenosine triphosphate (ATP), thereby facilitating energy supply to muscles and the brain. Arginine could promote cell proliferation, improve animal growth performance, and enhance immunity and antioxidant capacity [33-34]. The mean FPKM of DEG SARDH in the L-C group and L-60m group were 0.96 and 7.05, respectively, indicating that the expression response of this gene after stress was quite intense. Meanwhile, the expression level of DAM MW0107125 (C00581) also sharply increased in the ML-60 m group compared to ML-C group. The third ko00600 pathway showed that DEG LOC126997840 (GBA,srfJ) was a highly expressed gene in the untreated group (L-C), with an average FPKM of 117.93. However, in the 60 minute treatment group (L-60 m), the mean FPKM was as high as 716.67, indicating a significant change in the expression level of this gene after stress. The expression levels of DAM C00195, MW0111287 (C00836), MEDP1685 (C12144) and MW0055323 (C00550) co-expressed in this pathway also significantly increased after 60 minutes of treatment (Fig 7d). Phytosphingosine (C12144), as the precursor of ceramide (C00195), was a natural broad-spectrum antibacterial agent. Sphingomyelin (C00550) regulated immune function and inflammatory reaction, and participated in oxidative stress reaction [35]. Many cellular stress inducers, such as inflammatory activation, excessive saturated fatty acid intake, and chemotherapy, could lead to an increase in the rate of ceramide (C00195) synthesis [36-37].

**Discuss**

In this study, we integrated transcriptomics and metabolomics analysis of hepatopancreas and ovary, and looked for the regulatory mechanism of Eriocheir sinensis's adaptability to aflatoxin B1 from the perspective of differential expression of genes and metabolites. DEGs and DAMs revealed the differential response between groups without AFB1 and groups with AFB1, which helps us to analyze and manage this stress response. Transcriptomics results showed that a series of complex changes occurred in hepatopancreas and ovary within one hour after AFB1 injection. The hepatopancreas, as an immune and detoxifying organ, has a natural response to AFB1, as evidenced by changes in the expression of many functional genes. However, we did not find this strong response in the ovaries. We speculate that within one hour of being invaded by AFB1 in crabs, the hepatopancreas have already participated in various regulatory responses to cope with this stress, while the ovaries are not the main response organs during this period, and their responses are not very sensitive.

Compare the blank working solution groups (L-C) and standard AFB1 working solution groups (L-30 m and L-60 m) in hepatopancreas, and annotate the generated DEGs into GO, KEGG, and NR databases, respectively, to screen the target genes as the focus of this study. The hepatopancreas is the main detoxifying organ and the main site for material metabolism and transformation, making it highly susceptible to damage from toxic substances [3]. According to reports, AFB1 induced hepatopancreatic injury is mainly mediated by four mechanisms: CYP450 enzyme, mitochondria, immune system, and free radicals [38]. In our sequencing results, we found that the gene novel.6517 and the gene LOC126984017 (UGT) were associated with the synthesis of cytochrome P450. Gene novel.6517 encoded NADPH--cytochrome P450 reductase, with a mean FPKM of 0.01 and 2.50 in the L-C and L-60 m groups, respectively. The mean FPKM of gene LOC126984017 (UGT) in L-C group and L-30m group were 18.30 and 65.73, respectively, and it is involved in the metabolism of xenobiotics and drug by cytochrome P450. The abnormal increase in the expression levels of these two genes indirectly indicates that P450 enzymes are more active in response during stress.

Further systematic analysis showed there were 64 identical DEGs in the comparison between L-C vs. L-30 m and L-C vs. L-60 m, of which 34 identical DEGs were up-regulated. Based on functional annotation information and literature reports, we dissected 14 DEGs and classified them into six categories of functions, including antibacterial and detoxification, ATP energy reaction, redox reaction, nerve reaction, liver injury repair and immune reaction. According to reports, AFB1 induced immune toxicity reactions may lead to dysfunction of oxidative stress function [39]. During this process, we discovered two DEGs (LOC126981104, LOC126981425) associated with oxidative reactions and one DEG (LOC127005969) associated with immune responses, indicating that AFB1 can cause an increase in the expression of redox related genes, thus supporting the viewpoint reported in the above literature. In practical applications, antioxidants can be added to animal feed to protect and prevent AFB1 poisoning in livestock and poultry by enhancing antioxidant capacity and immune function [40]. The literature indicates that an increase in the expression levels of two DEGs (LOC126998993, novel.4226) indicates liver injury, indicating that these two genes have a self-evident repair effect on liver tissue [26]. The abnormal expression of four hydrolytic enzymes related to regulatory effects encoded by DEGs (LOC127004133, LOC127005812, LOC126997672, and LOC127004138) is related to the antibacterial and detoxification of the liver, and has also been mentioned in the literature [27-28]. Additionally, DEGs are also involved in neuromodulation and energy response, which are also synergistic reactions of hepatopancreas after stress.

The results of the metabolomics showed a total of 12 pathways were enriched in the comparison between ML-C vs. ML-30 m and ML-C vs. ML-60 m. Among them, Cyanoamino acid metabolism (ko00460), glutathione metabolism (ko00480), sulfur relay system (ko04122) and sphingolipid metabolism (ko00600) were significantly enriched (P<0.05). Two DAMs (MW0106760, MW0110603) were involved in the glutathione metabolism, which has been reported to have antioxidant, immune, and detoxifying properties effects [41]. Three DAMs (MW0111287, MW0055323, and MEDP1685) were involved in the sphingolipid metabolism, and sphingolipids have been reported to involve in many important signal transduction processes, including regulating cell growth, differentiation, aging, and programmed cell death [42]. From the above enrichment analysis results, it can be inferred that the expression of metabolites in hepatopancreas is also actively responding to AFB1 stress.

The Co-enriched pathways of metabolomics and transcriptomics were subjected to explore the interaction mechanism between them. The seven pathways Co-enriched by DEGs and DAMs between the untreated group (C) and the 30 min treat groups (30 m) and the nine pathways Co-enriched by DEGs and DAMs between the untreated group (C) and the 60 min treat groups (60 m) shared four identical pathways, including glycine, serine and threonine metabolism, tryptophan metabolism, tyrosine metabolism and purine metabolism. The metabolic enrichment of the above three amino acids reflects the synergistic response and consistent regulation of DEG and DAM when the hepatopancreas faces AFB1 stress. These feedbacks provide good research ideas and directions for future research on this stress regulation, especially in the discovery of important functions in the glycine, serine and threonine metabolism. DEGs and DAMs involved in this pathway are up-regulated, which will be further studied in detail in the following discussion. We anchored the up-regulated DEGs and DAMs on the KEGG map and depicted their interaction relationship on the map. Three interaction signals were generated from specific positions on the KEGG maps, including steroid biosynthesis (ko00100), glycine, serine and threonine metabolism (ko00260), and sphingolipid metabolism (ko00600).

In steroid biosynthesis, ergosterol is produced through the actions of DEG LOC127008729 (EGR3) and DAM MW0148978 (C05440), which are reaction enzyme and substrate on the KEGG map, respectively. Ergosterol is an important component of fungal cell membranes and pl/ays an important role in ensuring the integrity of membrane structure. Ergosterol can significantly inhibit tumor growth and activate inhibitory genes, therefore demonstrating a strong antibacterial ability. Research has shown that it can also enhance immune function and convert into fat soluble vitamin D2 [29-30]. In the metabolism of glycine, serine, and threonine, DEG LOC126981425 (SARDH) encodes sarcosine dehydrogenase, which is an important catalytic enzyme for the production of glycine. Glycine has anti-inflammatory and immunomodulatory effects, and literature has shown that it has physiological protective effects on liver injury and hepatitis [31-32]. The metabolite guanidinoacetate represented by MW0107125 (C00581) on KEGG map will ultimately be converted into creatine, which then converts into arginine and proline metabolites. According to reports, arginine can improve animal growth performance, enhance immunity and antioxidant capacity. Therefore, it is often used as a feed additive, with effects such as wound healing, ammonia excretion, immune function, and hormone secretion [33-34]. The synergistic up-regulation of LOC126981425 (SARDH) and MW0107125 (C00581) in this pathway, combined with their functions, can infer the regulatory response and protective mechanism played by the hepatopancreas in crabs. In sphingolipid metabolism, the metabolites dihydrosphingosine, phytosphingosine, sphingomyelin and ceramide were all detected as DAMs on the ko00600 map. They can transform into each other under certain conditions. phytosphingosine is produced as a natural broad-spectrum antibacterial agent; sphingomyelin and ceramide could regulate immune function, inflammatory reaction and oxidative stress reaction[35-37]. The function of these DAMs can also indicate an enhancement of hepatopancreatic sphingolipids metabolism to resist the invasion of AFB1.

This study explored genes or metabolites related to antibacterial activity, redox ability, liver injury protection, immune enhancement, and anti-inflammatory repair by combining target functional genes, metabolites, and metabolic pathways screened by multiple omics. These genes and metabolites play an important role in responding to the invasion of AFB1 in the hepatopancreas or ovaries of crabs. This experiment provides us with valuable insights into the molecular mechanism of the response of crab hepatopancreas to AFB1, and provides direction and ideas for future guidance in addressing AFB1 damage to crabs.

**Acknowledgements**

This Study was supported in part by the 333 High-level Talent Training Project of Jiangsu Province (BRA2019093), the Jiangsu Independent Innovation Project of Agricultural Science and Technology (CX(19)3007), and the Jiangsu Modern Agricultural Industry Technology System Project (JATS[2022]371).

**Conflict of interest**

The authors declare no conflict of interest exists.

**TABLES**

**Table 1** Data statistics of transcriptome sequencing

**Table 2** Up-regulated DEGs in comparison between L-C vs. L-30m and L-C vs. L-60m

**Table 3** Down-regulated DEGs in comparison between L-C vs. L-30m and L-C vs. L-60m

**Table 4** Cored and up-regulated DEGs during AFB1 injection in hepatopancreas

**Table 5** DEGs in comparison between R-C vs. R-30m and R-C vs. R-60m

**Table6** KEGG pathways were enriched by DAMs in ML-C vs. ML-30m comparison

**Table 7** KEGG pathways were enriched by DAMs in ML-C vs. ML-60m comparison

**Table 8** KEGG pathways were Co-enriched by DEGs and DAMs in C vs. 30 m comparison

**Table 9** KEGG pathways were Co-enriched by DEGs and DAMs in C vs. 60 m comparison

**Figure legends**

**Fig 1** PCA in hepatopancreas and ovary and DEGs in hepatopancreas. **(A)** PCA showed differences between the untreated groups (C) and the treated groups (30 m, 60 m) in hepatopancreas and ovary. **(B)** DEGs generated between C vs. 30 min and C vs. 60 min in hepatopancreas, as well as venns and heatmaps generated between C vs. 30 min and C vs. 60 min.

**Fig 2** Six categories of functions were classified by 14 DEGs, with Log2FC in C vs. 30 min and C vs. 60 min.

**Fig 3** DEGs generated in ovary. **(A)** Volcanic maps of DEGs generated by C vs. 30 min and C vs. 60 min in the ovaries. **(B)** venns and heatmaps generated between C vs. 30 min and C vs. 60 min in ovary.

**Fig 4** K-means analysis of hepatopancreas.

**Fig 5** Characteristics of metabolites and DAMs in hepatopancreas. **(A)** 25 major categories were classified by metabolites. **(B)** The OPLS-DA model showed significant differences between the untreated groups (C) and the treated groups (30 m, 60 m) in hepatopancreas. **(C)** Volcanic maps of DAMs generated by C vs. 30 min and C vs. 60 min in hepatopancreas.

**Fig 6** Enrichment analysis of KEGG pathway and Pearson correlation analysis of the top 50 differential metabolites in hepatopancreas and ovaries. **(A)** Enrichment analysis of KEGG pathway for C vs. 30 min in hepatopancreas. **(B)** Enrichment analysis of KEGG pathway for C vs. 60 min in hepatopancreas. **(C)** Enrichment analysis of KEGG pathway for C vs. 60 min in ovary. **(D)** Pearson correlation analysis of the top 50 differential metabolites for C vs. 30 min in hepatopancreas. **(E)** Pearson correlation analysis of the top 50 differential metabolites for C vs. 60 min in hepatopancreas. **(F)** Pearson correlation analysis of the top 50 differential metabolites for C vs. 60 min in ovary.

**Fig 7** KEGG pathways Co-enriched by DEGs and DAMs and interaction signals generated by up-regulated DEGs and DAMs. **(A)** KEGG pathways Co-enriched by DEGs and DAMs between the comparison of C vs. 30 min and C vs. 60 min in hepatopancreas. **(B)** Interaction signals at specific local locations in steroid biosynthesis pathway. **(C)** The interaction signals at specific local locations in glycine, serine and threonine metabolism. **(D)** The interaction signals at specific local locations in sphingolipid metabolism.

**Fig S1** The OPLS-DA model validation in ML-C vs. ML-30m comparison

**Fig S2** The OPLS-DA model validation in ML-C vs. ML-60m comparison

**References**

1. Bullerman L B. Food Technology, 1986, 40(5): 59-66]. Wang JS， Groopman JD. DNA damage by mycotoxins ［J］. Mutat Res， 1999， 424 （1-2）： 167-181.
2. Wang JS， Huang T， Su J， et al. Hepatocellular carcinoma and aflatoxin exposure in Zhuqing Village， Fusui County， People’s Republic of China ［ J］. Cancer Epidemiol Biomarkers Prev，2001， 10 （2）： 143-146.
3. Wang LY， Hatch M， Chen CJ， et al. Aflatoxin exposure and risk of hepatocellular carcinoma in Taiwan ［J］. Int J Cancer， 1996， 67（5）： 620-625
4. Schoenhard G L, Hendricks J D, Nixon J E, et al.Aflatoxicol-induced heatocellular carcinoma in rainbow trout (Salmo gairdneri) and the synergistic effects of cyclopropenoid fatty acids [J]. Cancer Research, 1981,41(3): 1011—1014
5. Chen H Y, Rawlings R. The truth of mycotoxin contamination of feed in Asia region [J]. China Poultry, 2018,30(16): 33-35.
6. Wang G Q, Liu Y D, Duan S H. Mycotoxin contamination and investigation of feed and feedstuff in the first half of 2019 [J]. Swine Production, 2019(6): 17-19
7. Deng S X, Tian L X, Liu F J, et al. Toxic effects and residue of aflatoxin B1 in tilapia (Oreochromis niloticus×O. aureus) [J]. Aquaculture, 2010, 307(3—4):233—240,
8. Ngethe S, Horsberg T E, Mitema E, et al. Species differences in hepatic concentration of orally administered 3H AFB1 between rainbow trout (Oncorhynchus mykiss) and tilapia (Oreochromis niloticus) [J]. Aquaculture, 1993,114(3—4): 355—358
9. Halver J E. Aflatoxicosis and trout hepatoma. In: Goldblatt L A (Eds.), Aflatoxin: Scientific Background, Control, and Implications [M]. New York: Academic Press.1969, 265—306;
10. Yu Y Y, Niu J, Yin P, et al. Detoxification and immunoprotection of Zn (II)-curcumin in juvenile Pacific white shrimp (Litopenaeus vannamei) feed with aflatoxin B1[J].Fish and Shellfish Immunology, 2018, 80: 480—486;
11. Plakas S M, Loveland P M, Bailey G S, et al. Tissue deposition and excretion of 14C-labeled aflatoxin B1 after oral administration in channel catfish [J]. Food and Chemical Toxicology, 1991, 29(12): 805—808,
12. Sahoo P K, Mukherjee S C. Immunosuppressive effects of aflatoxin B1 in Indian major carp (Labeo rohita) [J]. Comparative Immunology Microbiology and Infectious Diseases, 2001, 24(3): 143—149
13. El-Banna R, Teleb H M, Hadi M M, et al. Performance and tissue residue of tilapia fed dietary aflatoxin [J] Veterinary Medical Journal Giza, 1992, 40: 17—23.
14. Huang Y, Han D, Xiao X C, et al. Effect of dietary aflatoxin B1, on growth, fecundity and tissue accumulation in gibel carp during the stage of gonad development [J]. Aquaculture, 2014, 428—429(5): 236—242
15. Jantrarotai W, Lovell R T. Subchronic toxicity of dietary aflatoxin B1 to channel catfish [J]. Journal of Aquatic Animal Health, 1990, 2(4): 248-254;
16. Sahoo P K, Mukherjef S C, Jain A K, et al. Histopathological and electron microscopic studies of gills and opisthonephros of rohu, Labeo rohita to acute and subchronic aflatoxin B1 toxicity [J]. Asian Fisheries Science, 2003,16(3-4): 257-268;
17. Rui A G, Tu D C, Nguyen N T, et al. Aflatoxin B1 (AFB1) reduces growth performance, physiological response, and disease resistance in Tra catfish (Pangasius hypophthalmus) [J]. Aquaculture International, 2018,26(3): 921-936.
18. Zeng S L, Long W Q, Tian L X, et al. Effects of dietary aflatoxin B1 on growth performance, body composition, haematological parameters and histopathology of juvenile Pacific white shrimp (Litopenaeus vannamei) [J]. Aquaculture Nutrition, 2016, 22(5): 1152-1159.
19. NY5072-2002, Pollution-free food. Fishery limit with the feed safety [S]. Beijing: Ministry of Agriculture of the PRC, 2002.09.01.
20. TANG B P，K Y ZHOU，D X SONG，et a1．Molecular systematics of the Asian mitten crabs，genus Eriocheir (Crustacea：Brachyura)[J]．Molecular Phylogenetics and Evolution，2003，29(2)：309—316．
21. Wang, & W. (2004). A spiroplasma associated with tremor disease in the chinese mitten crab (eriocheir sinensis). Microbiology, 150(9), 3035-3040.
22. Daehwan, K., Ben, L., Salzberg, S.L., 2015. HISAT: A fast spliced aligner with low memory requirements. Nat. Methods 12 (4), 357–360.
23. Pertea, M., Pertea, G.M., Antonescu, C.M., Chang, T.C., Mendell, J.T., Salzberg, S.L., 2015. StringTie enables improved reconstruction of a transcriptome from RNA-seq reads. Nat. Biotechnol. 33 (3), 290–295.
24. He, C.; Liu, X.; Silva, J.A.T.D.; Liu, N.; Duan, J. Transcriptome sequencing and metabolite profiling analyses provide comprehensive insight into molecular mechanisms of flower development in Dendrobium officinale (Orchidaceae). Plant Mol. Biol. 2020, 104, 529–548
25. Gao, R. , Yong, Z. , & Gopalakrishnakone, P. . (2008). A novel trypsin-like serine proteinase from the venom of the chinese scorpion buthus martensii karsch. Ifmbe Proceedings, 21(1), 829-832.
26. El-Kabbani, et al. "Sorbitol dehydrogenase: structure, function and ligand design. " Current medicinal chemistry (2004).
27. Andri, J. , Lamata, M. , GMD Tejada, Bartels, R. , Koch, M. , & Brandenburg, K. . (2004). Cyclic antimicrobial peptides based on limulus anti-lipopolysaccharide factor for neutralization of lipopolysaccharide. Biochemical Pharmacology, 68(7), 1297-1307.
28. Milligan, S. , Martin, G. G. , Landrock, D. , Mcintosh, A. L. , Mackie, J. T. , & Schroeder, F. , et al. (2016). Impact of dietary phytol on lipid metabolism in scp2/scpx/l-fabp null mice. Biochimica et Biophysica Acta (BBA) - Molecular and Cell Biology of Lipids, 1862(3), 291-304.
29. Hashim, S. N., Schwarz, L. J., Danylec, B., Mitri, K., Yang, Y., & Boysen, R. I., et al. (2016). Recovery of ergosterol from the medicinal mushroom, ganoderma tsugae var. janniae, with a molecularly imprinted polymer derived from a cleavable monomer-template composite. Journal of Chromatography A,1468, 1-9.
30. K Krumpe, I Frumkin, Y Herzig, N Rimon, C Özbalci, & B Brügger, et al. (2012). Ergosterol content specifies targeting of tail-anchored proteins to mitochondrial outer membranes. Molecular Biology of the Cell, 23(20), 3927-3935.
31. Li, X. , et al. "Dietary Glycine Prevents Peptidoglycan Polysaccharide-Induced Reactive Arthritis in the Rat: Role for Glycine-Gated Chloride Channel." Infection and Immunity 69.9(2001):5883-5891.
32. Ikejima, et al. "Kupffer cells contain a glycine-gated chloride channel. " American Journal of Physiology (1997):7,272(6):1581-1586.
33. Scaglia, F. , et al. "Clinical consequences of urea cycle enzyme deficiencies and potential links to arginine and nitric oxide metabolism. " The Journal of Nutrition (2004).,134(10):775- 782.
34. Vissers, Y. L. , et al. "Plasma arginine concentrations are reduced in cancer patients: evidence for arginine deficiency?." American Journal of Clinical Nutrition 81.5(2005):1142-1146.
35. Sara Grassi, et al. Sphingosine 1-Phosphate Receptors and Metabolic Enzymes as Druggable Targets for Brain Diseases. Front Pharmacol. 2019 Jul 23;10:807.
36. Kinga Czubowicz, et al. The Role of Ceramide and Sphingosine-1-Phosphate in Alzheimer's Disease and Other Neurodegenerative Disorders. Mol Neurobiol. 2019 Aug;56(8):5436-5455.
37. Maria Grazia Cattaneo, et al. Cross-talk between sphingosine-1-phosphate and EGFR signaling pathways enhances human glioblastoma cell invasiveness. FEBS Lett. 2018 Mar;592(6):949-961.
38. [1]Giri, et al. "Wobble dC·dA Pairing 5′ to the Cationic Guanine N7 8,9-Dihydro-8-(N7-guanyl)-9-hydroxyaflatoxin B[sub 1] Adduct: Implications for Nontargeted AFB[sub 1] Mutagenesis. " Biochemistry (2003):42:7023-7034.
39. Chen, J. , et al. "Effects of aflatoxin B1 on oxidative stress markers and apoptosis of spleens in broilers." Toxicology & Industrial Health (2016):278.
40. Ni-Ya, Zhang , et al. "Curcumin Prevents Aflatoxin B1 Hepatoxicity by Inhibition of Cytochrome P450 Isozymes in Chick Liver." Toxins 8.11(2016):327.
41. Grażyna Świderska-Kołacz, J. Klusek , and Adam Kołątaj. "The effect of exogenous GSH, GSSG and GST-E on glutathione concentration and activity of selected glutathione enzymes in the liver, kidney and muscle of mice." Animal Science Papers & Reports 25.2(2007):111-117.

van Kruining, DaanLuo, Qianvan Echten-Deckert, GerhildMielke, Michelle M.Bowman, AndrewEllis, ShaneOliveira, Tiago GilMartinez-Martinez, Pilar. "Sphingolipids as prognostic biomarkers of neurodegeneration, neuroinflammation, and psychiatric diseases and their emerging role in lipidomic investigation methods." Advanced drug delivery reviews 159(2020):232-244.
