# Supplementary material for: Transcriptome and metabolomics analysis of adaptive mechanism of Chinese mitten crab (Eriocheir sinensis) to aflatoxin B1
Source: PLoS One. 2023 Dec 7;18(12):e0295291. doi: 10.1371/journal.pone.0295291 (PMC10703319; doi:10.1371/journal.pone.0295291)
Supplement: Attachment — Submitted filename: Response to Reviewers.docx [file pone.0295291.s012.docx]

Journal Requirements:

When submitting your revision, we need you to address these additional requirements.

1. Please ensure that your manuscript meets PLOS ONE's style requirements, including those for file naming. The PLOS ONE style templates can be found at

https://journals.plos.org/plosone/s/file?id=wjVg/PLOSOne_formatting_sample_main_body.pdf and

https://journals.plos.org/plosone/s/file?id=ba62/PLOSOne_formatting_sample_title_authors_affiliations.pdf

**We have made the necessary modifications according to the instructions.**

2. We note that the grant information you provided in the ‘Funding Information’ and ‘Financial Disclosure’ sections do not match.

When you resubmit, please ensure that you provide the correct grant numbers for the awards you received for your study in the ‘Funding Information’ section.

**We delete the ‘Funding Information’, and have received no specific funding for this work.**

3. Thank you for stating the following in the Acknowledgments Section of your manuscript:

“This Study was supported in part by the 333 High-level Talent Training Project of Jiangsu Province (BRA2019093), the Jiangsu Independent Innovation Project of Agricultural Science and Technology (CX(19)3007), and the Jiangsu Modern Agricultural Industry Technology System Project (JATS[2022]371).”

We note that you have provided funding information that is not currently declared in your Funding Statement. However, funding information should not appear in the Acknowledgments section or other areas of your manuscript. We will only publish funding information present in the Funding Statement section of the online submission form.

Please remove any funding-related text from the manuscript and let us know how you would like to update your Funding Statement. Currently, your Funding Statement reads as follows:

“The author(s) received no specific funding for this work.”

Please include your amended statements within your cover letter; we will change the online submission form on your behalf.

**We have modified the acknowledgment section and added this sentence to the cover letter,“The author(s) received no specific funding for this work.”**

4. PLOS requires an ORCID iD for the corresponding author in Editorial Manager on papers submitted after December 6th, 2016. Please ensure that you have an ORCID iD and that it is validated in Editorial Manager. To do this, go to ‘Update my Information’ (in the upper left-hand corner of the main menu), and click on the Fetch/Validate link next to the ORCID field. This will take you to the ORCID site and allow you to create a new iD or authenticate a pre-existing iD in Editorial Manager. Please see the following video for instructions on linking an ORCID iD to your Editorial Manager account: https://www.youtube.com/watch?v=_xcclfuvtxQ

**We create a ORCID iD for the corresponding author. ID number: 0000-0002-8595-5783.**

5. We are unable to open your Supporting Information file [Supporting Information.rar and Figures.rar]. Please kindly revise as necessary and re-upload

**We have uploaded each figure and table separately.**

6. Please include a copy of Tables 1-9 which you refer to in your text on page 27.

**Sorry, the Tables were in the compressed package,** [**Supporting Information rar.**] **We have re uploaded the tables.**

7. Please upload a copy of Figures 1-7, to which you refer in your text on page 28. If the figure is no longer to be included as part of the submission please remove all reference to it within the text.

**Sorry, the Figures were in the compressed package, [Figures.rar.] We have re uploaded the Figures.**

8. Please review your reference list to ensure that it is complete and correct. If you have cited papers that have been retracted, please include the rationale for doing so in the manuscript text, or remove these references and replace them with relevant current references. Any changes to the reference list should be mentioned in the rebuttal letter that accompanies your revised manuscript. If you need to cite a retracted article, indicate the article’s retracted status in the References list and also include a citation and full reference for the retraction notice.

**Not modified**

To Reviewer #1

1. Grammatical and spelling mistakes

**we check it and correct it**

2. Scientific names should be in italics

species names, genes and other scientific names were italics in this revision.

**Corrected.**

3. Please change ul-µl throughout the manuscript

**Corrected.**

4. Comparison data, results, or references should be there in the Results section. Statistical data should be explained more properly

**I'm sorry, I uploaded the figures and tables of the results to the website in a compressed package. However, for some reason, this file cannot be seen by you. This submission will upload each data result separately.**

5. Line 48- Food and feed?

**It mainly present in feed, delete “ food”.**

6. Line 58 and 59- Rewrite the sentence

**The effects of AFB1 on aquatic animals have been widely reported in many research articles both domestically and internationally, such as *Oreochromis niloticus* [7,8], *Oncorhynchus mykiss* [9], *Litopenaeus vannamei* [10], *Lctalurus punctatus* [11], *Labeo rohita* [12] and other species.**

7. Line 81- We hope? It is research work and if results are there then why expect?

**This sentence is not appropriate. We have changed it to “ we analyzed the obtained data in this experiments and explained some molecular response mechanism in the results.**

8. Line 96 and Line 101- “certain amount”- Please mention the amount

**Line 96: by weighing 6 mg of aflatoxin B1 standard and dissolving it with 100 ml of dimethyl sulfoxide**

**Line 101 : “More than 18 female experimental crabs”**

9. Line 107-Line 109- After 30 minutes or 60 minutes? Give only one-time point that was done for the experiment

**I rewrote this sentence. “The hepatopancreas and ovaries of experimental crabs injected with AFB1 in first group were taken out after 30 minutes, and those in the second group were taken out after 60 minutes”**

10. Line111- “Frozen samples with liquid nitrogen”- Rewrite the sentence

**The samples were frozen with liquid nitrogen and quickly stored in an ultra-low temperature refrigerator (-80℃)**

11. Line 126- Different groups- Mention the groups

**Correct into “the above three groups (one control and two treatments)”**

12. Line 139-Line 141-Rewrite the sentence-It is not clear

**The clean reads were aligned to the reference genome *Chinese mitten crab* (*Eriocheir sinensis)* (https://www.ncbi.nlm.nih.gov/assembly/GCF_024679095.1) using HISAT 2 software**

13. Line 149-Line 150- Rewrite the sentence-Not clear

**I rewrote this sentence: the screening criteria for DEGs was FDR < 0.05 and fold change ≥ 2.**

14. Line 170-Line 173- Explain the statistical analysis in detail

**I rewrote this sentence “The differential metabolites were screened by combining the differential multiple, P value of the t-test and VIP value of the OPLS-DA model, and the screening standard was FC > 1, P value < 0.05 and VIP > 1. ” T-test is the use of t-distribution theory to infer the probability of differences occurring. The screening of differential metabolites is usually based on the OPLS-DA model, and VIP is used to evaluate the importance of each metabolite in metabolomic data analysis of sample classification or prediction models, usually greater than 1.**

15. Line 191- PC1 and PC2- What are these? Explain?

**Principal Component Analysis (PCA) is the most widely used data dimensionality reduction algorithm. All features of the sample are reflected using two principal components, PC1 and PC2. The position relationship of samples on the coordinate axis reflects the similarity between samples. The closer the position is, the higher the similarity between the two samples. The numerical value represents the explanatory rate of variance, and the larger the numerical value, the more reliable the model is.**

16. Line 201- Line 205- Break the sentences to make it more clear

**I rewrote this sentence “In hepatopancreas and ovaries，AFB1 for 30 minutes and 45 minutes groups were compared with control group, respectively.”**
